# Effects of HIIT and MICT on cardiovascular risk factors in adults with overweight and/or obesity: A meta-analysis

**DOI:** 10.1371/journal.pone.0210644

**Published:** 2019-01-28

**Authors:** LiQiang Su, JinMei Fu, ShunLi Sun, GuangGao Zhao, Wei Cheng, ChuanChuan Dou, MingHui Quan

**Affiliations:** 1 School of Physical Education and Sport Science, Fujian Normal University, Fuzhou, Fujian, China; 2 Jiangxi Research Institute of Sports Science, Nanchang, Jiangxi, China; 3 Department of Physical Education, Nanchang University, Nanchang, Jiangxi, China; 4 Department of Endocrinology, Yangpu Hospital Affiliated to Tongji University, Shanghai, China; 5 College of Humanities, Jiang Xi University of Traditional Chinese Medicine, Nanchang, Jiangxi, China; 6 School of Kinesiology, Shanghai University of Sport, Shanghai, China; UNSW Sydney, AUSTRALIA

## Abstract

**Objective:**

The purpose of this study was to evaluate the effects of high-intensity interval training (HIIT) and moderate-intensity continuous training (MICT) on cardiovascular disease (CVD) risk factors in adults with overweight and obesity.

**Methods:**

Twenty-two articles were included by searching six databases, the total number of subjects was 620 in these articles. Outcomes were synthesised using a random-effects meta-analysis of the Standardized mean difference (SMD) in CVD risk factors.

**Results:**

HIIT and MICT resulted in statistically significant reductions in Weight, BMI, fat%, total cholesterol(TC), and improvement in VO_2max_. Compared with MICT, subgroup of durations of HIIT training interval ≥2 min can significantly increase VO_2max_ (SMD = 0.444, 95% CI:0.037~0.851,*P* = 0.032), subgroup of energy expenditure of HIIT equal to MICT can significantly increase VO_2max_ (SMD = 0.399, 95% CI:0.106~0.692,*P* = 0.008).

**Conclusions:**

HIIT appears to provide similar benefits to MICT for improving body composition, VO_2max_and TC, but HIIT spent less time than MICT by 9.7 min on one session. HIIT is superior to MICT in improving cardiopulmonary fitness when durations of HIIT training interval ≥2 min or energy expenditure of HIIT same as MICT. PROSPERO ID: CRD42016045835.

## Introduction

According to epidemiological data, cardiovascular disease (CVD) is the leading cause of death and disability[1, 2]. People who develop CVD not only bear extreme suffering leading to declines in quality of life but also pose a heavy economic burden to their families and the society. BMI and waist circumference are correlated with risk for CVD, its risk factors, and overall mortality,additionallyweight loss medications, but not behavior-based interventions, were associated with higher rates of harms[3]. Therefore, it is of practical importance to identify an appropriate method, especially an no-drug method, of improving the risk factors of CVD and reducing its incidence. Moderate-intensity continuous training (MICT) is often considered an effective way to reduce risk factors of CVD [4, 5]. However, in recent years, a popular protocol calledhigh-intensity interval training (HIIT) which involved brief high-intensity by rest or brief but slightly longer bouts of very low-intensity exercise, is one of the most effective means of improving cardiorespiratory and reducing risk factors of CVD [6–8]. Therefore, a body of experimental studies have been conducted to compare the effects between HIIT and MICT, but their results are inconsistent and inconclusive. Indeed, some experimental studies reported HIIT were more effective than MICT in reducing risks of CVD[9–11], but other studies did not find this result[12]. Although previous meta-analyses have been conducted to compare the effects between two training methods in adolescents with abnormal glucose and lipid metabolism[13, 14], the results HIIT vs. MICT for reducing risk factors of CVD are inconsistent[11, 15–19]. Therefore, we aimed to quantitatively combine data from experimental studies compare the effect between HIIT and MICT in reducing risk factors of CVD in adults with overweight and/or obesity. Findings from this study may serve important evidence for prevention CVD in adults with overweight and/or obesity.

## Methods

### Literature search

This research program has been registered on the PROSPERO System Evaluation Registration Platform, registration number: CRD42016045835. This study has been reported according to the preferred reporting items for systematic reviews and meta-analyses (PRISMA) guidelines. The searched databases included PubMed, Embase, Cochrane, CENTRAL, PEDro and CNKI. The search strategy comprised key phrases ’interval training’ OR ’interval exercise’ OR ’intermittent exercise’ OR’ intermittent training’ ADN ’overweight’ OR ’obesity’ OR ’obese’ to identify relevant trials, the search strategy was limited to adults, and not animals, adolescent, child. The publication dates of the articles were restricted to the period between the year when the database was built to July 20, 2018.

### Study inclusion and exclusion criteria

Inclusion criteria were as follows: (a) randomized controlled trial (RCT) or controlled clinical trial (CCT); (b) healthy participants ≥ 18 years old, BMI ≥ 25; (c) HIIT intervention and MICT intervention,HIIT defined as activities with intermittent bouts of activity that were performed at maximal effort, ≥75% VO_2max_, ≥75% HR reserve or the relative intensity of at least 85% HR _max_, The study included a HIIT session lasting ≤4 min/set interspersed with an interval of rest or active recovery; (d) ≥ 4 weeks with intervention; (e) outcome indicators included at least one of the following: weight, body mass index (BMI), fat mass% (fat%), VO_2max_, triglyceride (TG), total cholesterol (TC), low-density lipoprotein (LDL), high-density lipoprotein (HDL), fasting blood glucose (FBG) and insulin; (f) the study reported that baseline BMI of HIIT participants or baseline BMI could be calculated from the provided data; (g) the article was written in English or Chinese. Exclusion criteria were as follows: (a) participants with chronic diseases, such as diabetes, hypertension, or other diseases constituting a special population; (b) studies on one-time acute exercise; (c) interventions including strength training, diet or medicine; (d) HIIT without supervision.

### Data collection

Two authors independently screened titles and abstracts of potentially eligible studies and downloaded the full texts. Discrepancies between the two authors during the literatures exclusion were solved through reaching consensus with each other. Another way of obtaining eligible studies was through examining the references of relevant studies.

### Literature quality evaluation

The quality of the literature was evaluated according to the risk bias evaluation method adopted by Costigan et al. [14] in a meta-analysis of HIIT-related research. The eight evaluation items were as follows: (a) inclusion criteria; (b) randomized grouping; (c) baseline similarity; (d) rater-masked; (e) intentional analysis; (f) participants’ withdrawal proportion was less than 20%; (g) the sample quantity met the requirements; (h) accurate results were reported. The two researchers used ’’√’’ (with clear description), "×" (without description) and "?" (unknown or inadequate description) to evaluate each included article. The inconsistent results were solved through discussion among the research group, and each article with a ’’√’’ was counted.

### Statistical treatment

Given the consistency of variable units between the same outcome indicator among the continuous variables in the included studies, we compare the changes from baseline to end-point data between groups. Some SD of change is sometimes not supplied, based on Cochrane Handbook for Systematic Reviews of Interventions, we calculate the correlation coefficients using a complete report of the study, and use the correlation coefficients to estimate the SD of the changes from baseline to end-point. Within-group meta-analyses were completed for continuous data using the baseline and post-intervention values. Random-effects models were used in this study for the meta-analysis of included studies. Standardized mean difference(SMD)and 95% CI were calculated in this study for statistical analysis. According to the characteristics of the literature, the factors of durations of HIIT training interval and energy expenditure were analyzed to test the effects of different subgroups. The statistical heterogeneity was examined using I^2^ between included studies and Cochran’s Q-test. It was defined as non-existent, low, medium and high heterogeneity when I^2^ values were < 25%, 25 ~ < 50%, 50 ~ < 75%, and ≥ 75%, respectively[19]. Egger test was adopted to detect publication bias. When there was a significant Egger test for publication bias (*P* <0.05), the trim and fill method was performed to estimate the impact of publication bias on the results[20]. Furthermore, to test the reliability of the results of this study, the following two methods were used to conduct a sensitivity analysis: one article was removed each time to examine whether each article had a significant influence on the effect. Level of significance was set at *P*< 0.05 and 95% confidence intervals. Magnitude of effect was categorized as large (SMD > 0.8), medium (SMD 0.5~0.8), small (SMD 0.2~0.5) or trivial(SMD < 0.2)[21]. All statistical calculations were performed by statistical software STATA 14.1 (Release 14.1 College Station, TX, USA); *P* < 0.05 was defined as a significant difference.

### Subgroup moderator analysis

HIIT must be applied appropriately by manipulating key programming variables (frequency, intensity, training interval, recovery interval). There are difference contributions of the anaerobic energy systems during events of differing training interval and intensities. The phosphagen and fast glycolytic are the primary energy systems when durations of HIIT training interval < 2 min (DHTI < 2 min), the fast glycolytic energy systems will be gradually depleted when durations of HIIT training interval ≥ 2 min (DHTI ≥2 min)[22]. HIIT programmes were divided into the following subgroups: DHTI ≥ 2 min and DHTI < 2 min. Energy expenditure values were included if reported in the manuscript or were calculated using reported data for group mean VO_2max_ and protocol intensity/duration assuming a 21 kJ min^-1^ energy expenditure during exercise at a VO_2_ of 1 L min^-1^[19]. When exercise energy expenditure (EE) was reported or can be estimated by reported data, the studies were classified as HIIT equal to MICT (H = M), HIIT less than MICT (H<M) or HIIT more than MICT (H>M). On the contrary, when exercise EE was not reported and cannot be estimated, such as sprint interval protocols that have a large anaerobic component, the studies were classified as Not Clear (?). A*Z*-test was performed to compare the mean effect across subgroups.

## Results

### Literature search and selection

**Fig 1:**
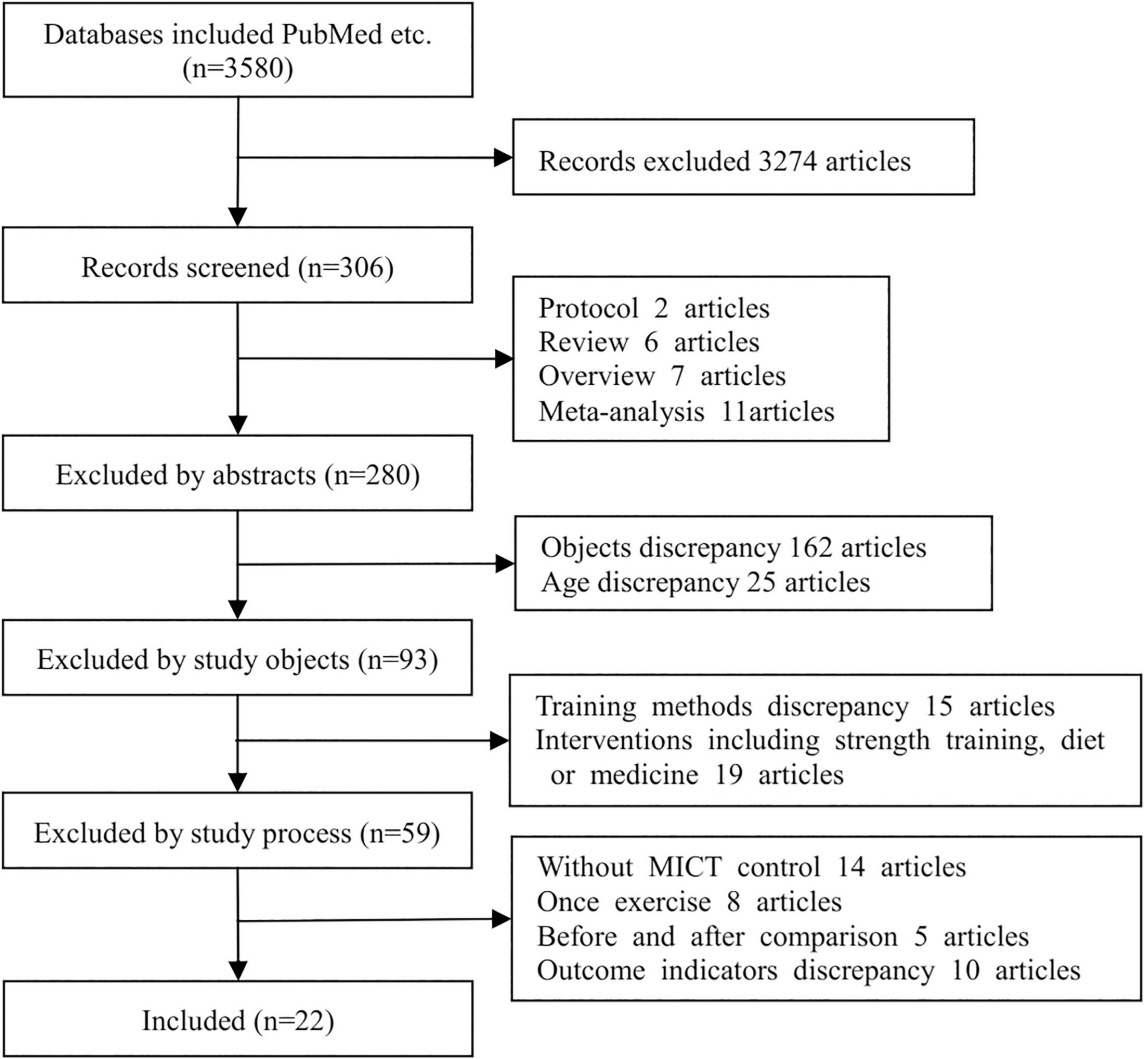
Study selection process.

A total of 3580 articles were searched from each database; 3274 articles were excluded according to the inclusion and exclusion criteria, and 22 articles were included[23–44]. The literature screening process is shown in Fig 1.

### Literature characteristics

There were 620participants in the 22 articles examined in this study. A total of 310subjects were in the HIIT group, with 6 ~ 29 subjects in each group; a total of 310 subjects were in the MICT group, with 7 ~ 29 subjects in each group. The training frequency was 3 ~ 5 times a week, and the cycle was 4 ~ 12 weeks. The participants included in this study were trained by ergometry cycle [24, 26, 28, 31–34, 36–38, 41], walking, jogging and running [18, 25, 27, 29, 30, 35, 39, 40, 42–44]. The following target intensity was used in the MICT group: 60 ~ 75% HR_max_[23, 26, 31, 32, 35], 50 ~ 60% VO_2max_[25, 30, 36, 41–43], 10% below the individual anaerobic threshold (IAT) intensity [24], 400% MET (metabolic equivalent of energy) [29], 60 ~ 80 VO_2peak_ [26, 28, 33, 34], 50 ~ 75% HR_peak_[40, 44]and 55 ~ 70%HRR[5, 38]. The following target intensity was used in HIIT group: 85 ~ 95%HR_max_ [23, 27, 29, 31, 35], 85 ~ 90% VO_2max_ [25, 30, 36, 41–44], all out[27, 32, 34, 37–39, 41], 85% peak power [28], 85 ~ 95% HR_peak_[40], 20% above the IAT intensity[24], 200% W_max_[33]and 120% VO_2peak_ [26]. VO_2max_ was determined by the method of the stepwise increasing load test combined with gas metabolic analysis. The standards of the end of exercise were that the respiratory quotient was more than 1.05 [23, 32], 1.1 [34, 38, 40]or 1.15 [25, 27, 30], combined with subjective consciousness and the exhaustion of willpower[29, 31, 36]. Blood collection after fasting 8 ~ 12 hours was needed for biochemical index detection. The methods adopted for the body composition test included the following: dual X-ray[23, 25, 26, 28, 31–33, 36, 37], bioelectrical impedance [24, 30, 34, 40–44], air displacement plethysmography with measured thoracic gas volume[38], and skin fold calculation[29]. The outcome indexes included body composition (weight, BMI, fat%), VO_2max_ and glycolipid metabolism indicators (TG, TC, LDL, HDL, FBG, insulin). Total duration of HIIT and MICT were 10.3±3.0 weeks, one session of HIIT and MICT was 30.0±10.2min and 39.7±15.5min respectively, The included studies’ characteristics are shown in Table 1.

**Table 1:** Summary of included studies.

Study	Age	BMI	Modality	Energy expenditure	Duration	MICT group				HIIT group				
						n	Intensity	Time(min)	Frequency	n	High intensity	Recovery intensity	Time(min)	Frequency
Schjerve et al.[23]	46.9	36.7	walking and running	H<M	12w	13	60–70% HR_max_	47	3/w	14	4×4-min 85–95% HR_max_	3 min 50–60% HR_max_	28	3/w
Moreira et al.[24]	40	28.3	cycle	H = M	12w	8	10% lower than IAT	60	3/w	8	2 min intensity was 20% above IAT	1 min 20% lower than IAT	60	3/w
Sijie et al.[25]	19.8	27.7	walking and jogging	H = M	12w	16	50% VO_2max_	40	5/w	17	5×3 min 85% VO_2max_	3 min 50% VO_2max_	30	5/w
Keating et al.[26]	41.8	28.2	cycle	?	12w	13	65% VO_2peak_	45	3/w	13	6×60s 120% VO_2peak_	120s low intensity	18	3/w
Lunt et al.-1[27]	48.2	32.1	walking and jogging	H = M	12w	7	65–75% HR_max_	33	3/w	9	4×4-min 85–95% HR_max_	3 min 65–75% HR_max_	28	3/w
Lunt et al.-2[27]	50.3	32.4	walking and jogging	?	12w	7	65–75% HR_max_	33	3/w	9	3×30sce all out	4 min low intensity	13.5	3/w
Wang et al.[40]	21	25.8	walking and jogging	H = M	12w	12	60–70% HR_peak_	33	4/w	12	4×4 min 85–95% HR_peak_	3 min 50–60% HR_peak_+7 min pacing rest	28	4/w
Fisher et al.[28]	20	29	cycle	H<M	6w	10	55–65% VO_2peak_,138±13w	60	5/w	13	4×30s 85% peak power,810±250w	4 min 15% peak power,140±20w	20	3/w
Cheema et al.[29]	43	32	walking	H = M	12w	6	4 MET	50	4/w	6	10×2 min >75% HR_max_	1 min, standing or pacing	30	4/w
Ahmadizad et al.[30]^#^	25	27.6	walking and jogging	?	6w	10	50–60% VO_2max_	60	3/w	10	8×2–3 min (rest/intensity = 2:1), intensity: 90% VO_2max_		24	3/w
Sawyer et al.[31]	35.1	37.4	cycle	H<M	8w	9	70–75% HR_max_	30	3/w	9	10×1 min 90–95% HR_max_	1 min low intensity	20	3/w
Martins et al.-1[32]	34.4	32.4	cycle	H = M	12w	7	70% HR_max_	250Kcal deficit time	3/w	16	8sce all out	12s low intensity	250Kcal deficit time	3/w
Martins et al.-2[32]	34.4	32.4	cycle	H<M	12w	7	70% HR_max_	250Kcal deficit time	3/w	16	8sce all out	12s low intensity	125Kcal deficit time	3/w
Cocks et al.[33]	25	35.8	cycle	?	4w	8	65% VO_2peak_	60	5/w	8	4–7×30s 200%W_max_	120s 30 W	30	3/w
Kong et al.[34]	21	25.8	cycle	H<M	5w	13	60–80% VO_2peak_	40	4/w	13	60×8s all out	12s passive rest	20	5/w
Gerosa-Neto et al.[35]	46.4	31.8	walking and running	H<M	16w	11	70% HR_max_	30	5/w	11	4×4 min90%HR_max_	3 min 75% HR_max_	28	3/w
Zhang et al.[36]	18–22	38.1% (fat%)	cycle	H = M	12w	15	60% VO_2max_	62.6	3-4/w	15	4–6×4 min 90% VO_2max_	3 min passive rest	34	3-4/w
Liu et al.[41]	20–23	28.8	cycle	H = M	12w	20	50% VO_2max_	30	4/w	20	15×1 min 90% VO_2max_	1 min 20% VO_2max_	30	4/w
Zhang et al.[39]	21	25.8	running	H = M	12w	12	60–70% HR_peak_	33	4/w	12	4×4 min 85–95% HR_peak_	3-min 50–60% HR_peak_	28	4/w
Higgins et al.[37]	20.4	30.3	cycle	H = M	6w	20	60%–70% HRR	20	3/w	29	(3–7)30s all out	4 min of active recovery	16	3/w
Vella et al.[38]	26.2	31.6	treadmill, cycle, elliptical	H = M	8w	9	55–59% HRR	20	4/w	8	10×1min 75–80% HRR	1min 35–40% HRR	20	4/w
Wang et al.[42]	18–21	28.7	treadmill, cycle	H = M	12w	16	60% VO_2max_	45	4/w	18	7×3min 80–90% VO_2max_	3min 50–60% VO_2max_+1min rest	36	4/w
Gao et al.[43]	21.6	27.1	treadmill	H = M	12w	17	60% VO_2max_	55	5/w	17	5×4min 85% VO_2max_	2min 50%VO_2max_+5min rest	55	5/w
Eimarieskandari et al.[44]	22.1	29.6	treadmill	H = M	8w	7	50–70% HR_peak_	41	3/w	7	4×4min80-90%VO_2peak_	3min50-60%VO_2peak_	33	3/w

MICT:moderate-intensity continuous training; HIIT:high-intensity interval training; IAT: individual anaerobic threshold; MET: Metabolic Equivalent; HR:heart rate; HRR:heart rate reserve. Lunt et al.-1 and Lunt et al.-2 were two different HIIT methods were used in one article. Martinset al.-1 and Martins et al.-2 were two different HIIT methods were used in one article. H = M: Energy expenditure of HIIT equal to MICT; H<M: Energy expenditure of HIIT less than MICT; ?:Not Clear; ^#^: the study is controlled clinical trial and non-randomised trials, unmarked studies arerandomized controlled trial.

### Bias and sensitivity analysis

Of the 22 articles, 5 articles were classified as low risk (risk of bias assessment ranging from 7 ~ 8), 16 articles were classified as moderate risk (risk of bias assessment ranging from 4 ~ 6), and one article was classified as high risk (risk of bias assessment ranging from 0 ~ 3), Risk of bias assessment of included studies are presented in Table 2. According to the sensitivity analysis of the 22 articles, if statistical models were replaced and one article was removed each time to perform a meta-analysis again, the results of effect would not change meaningfully, indicating that the results of the meta-analysis in this study were reliable. Egger test for fat% and Insulin determined indication of publication bias, the trim and fill method was proformed to fat% and the rusults suggested that 5 studies were missing, before trim and fill(SMD:-0.159, 95%CI: -0.509~0.191), after trim and fill(SMD: 0.642, 95%CI: 0.443~0.930), these values were changed after using the trim and fill method under the random-effects model, it suggestes that publication bias has great influence on the stability of fat%. The same method suggests that 2 studies were missing to Insulin, these values were unchanged using the trim and fill method under the random-effects model, it suggestes that the effect of publication bias on Insulin can be ignored. Egger test show no significant publication bias for other of the outcome variables(Table 3).

**Table 2:** Risk of bias assessment of included studies.

Article	Evaluation items								Score
	Inclusion criteria	Randomized grouping	Baseline similarity	Rater-masked	Intentional analysis	Withdrawal proportion is less than 20%	Sample quantity meets the requirements	Accurate results report	
Schjerve et al.[23]	√	√	√	√	?	√	×	√	6
Moreira et al.[24]	√	√	√	?	?	√	×	√	5
Sijie et al.[25]	√	√	√	?	?	√	×	√	5
Keating et al.[26]	√	√	√	√	√	√	×	√	7
Lunt et al.[27]	√	√	√	√	√	√	√	√	7
Wang et al.[40]	√	√	√	?	?	?	×	×	3
Fisher et al.[28]	√	√	√	√	√	√	×	√	7
Cheema et al.[29]	√	√	√	√	√	√	×	√	7
Ahmadizad et al.[30]	√	?	√	?	?	√	×	√	4
Sawyer et al.[31]	√	√	√	√	?	√	√	√	7
Martins et al.[32]	√	√	√	?	?	√	×	√	5
Cocks et al.[33]	√	√	√	?	?	√	×	√	5
Kong et al.[34]	√	√	√	?	?	√	√	√	6
Gerosa-Neto et al.[35]	√	√	√	?	?	√	×	√	5
Zhang et al.[36]	√	√	√	?	?	√	×	√	5
Liu et al.[41]	√	√	√	?	?	√	×	×	4
Zhang et al.[39]	√	√	√	√	×	√	×	√	6
Higgins et al.[37]	√	√	√	?	×	√	√	√	6
Vella et al.[38]	√	√	√	?	?	√	×	√	5
Wang et al.[42]	√	√	√	?	×	√	×	√	5
Gao et al.[43]	√	√	√	?	×	√	×	√	5
Eimarieskandari et al.[44]	√	√	√	?	×	√	×	√	5

√: with clear description; ×: without description; ?: unknown or inadequate description.

**Table 3:** Effects of HIIT vs. MICT on body composition, aerobic capacity, lipid metabolism, glucose metabolism.

Category	Index	Study (n)	Within-group effects				Between-group effects			
			HIIT		MICT					
			SMD (95% CI)	*P*	SMD (95% CI)	*P*	SMD (95% CI)	*P*	*I*^*2*^(%)	Egger test *P*
Body composition	Weight, kg	20	0.305(0.117, 0.494)	0.002*	0.319(0.129, 0.508)	0.001*	-0.039(-0.346, 0.268)	0.803	64.2	0.529
	BMI, kg/m^2^	16	0.591(0.139, 1.044)	0.010*	0.727(0.272, 1.182)	0.002*	0.017(-0.415, 0.449)	0.938	74.9	0.614
	fat%	18	0.609(0.248, 0.971)	0.001*	0.647(0.315, 0.979)	0.000*	-0.159(-0.509, 0.191)	0.374	68.4	0.016^§^
Aerobic capacity	VO_2max_, ml/kg/min	16	-0.966(-1.290, -0.642)	0.000*	-0.690(-1.037-, 0.343)	0.000*	0.256(-0.019, 0.531)	0.068	43.9	0.990
Lipid metabolism index	TG, mg/dl	12	0.146(-0.098, 0.390)	0.241	0.068(-0.180, 0.317)	0.589	-0.099(-0.489, 0.291)	0.619	60.4	0.536
	TC, mg/dl	12	0.467(0.157, 0.777)	0.003*	0.488(0.176, 0.800)	0.002*	0.121(-0.203, 0.445)	0.464	43.1	0.279
	LDL, mg/dl	8	0.445(0.092, 0.797)	0.013*	0.287(-0.086, 0.661)	0.132	-0.101(-0.376, 0.173)	0.470	0.0	0.274
	HDL, mg/dl	19	0.129(-0.204, 0.462)	0.447	0.031(-0.276, 0.339)	0.842	-0.092(-0.515, 0.332)	0.671	59.9	0.079
Glucose metabolism index	FBG, mmol/l	10	0.372(-0.401, 1.145)	0.345	0.344(-0.246, 0.934)	0.253	-0.139(-0.803, 0.524)	0.681	81.4	0.476
	Insulin, mU/l	7	0.585(-0.318, 1.488)	0.204	0.779(-0.063, 1.621)	0.070	0.348(-0.105, 0.801)	0.132	43.3	0.039^§^

*P<0.05 was defined as a significant difference;

^§^*P*<0.05 was defined as publication bias

MICT: moderate-intensity continuous training; HIIT: high-intensity interval training; SMD: Standardized mean difference; BMI: Body Mass Index; TG: triglyceride; TC: Total Cholesterol; LDL: Low-density lipoprotein; HDL: High-density lipoprotein; FBG: fasting blood glucose.

### Results of meta-analysis

Within-group analyses are showed in Table 3. Both HIIT and MICT resulted in statistically significant reductions in Weight (SMD: 0.305 and 0.319 for HIIT and MICT, respectively), BMI(SMD:0.591 and 0.727 for HIIT and MICT, respectively), fat%(SMD:0.609 and 0.647 for HIIT and MICT, respectively), TC(SMD:0.467 and 0.488 for HIIT and MICT, respectively), and improvement in VO_2max_(SMD:-0.966 and -0.690 for HIIT and MICT, respectively), additionally, HIIT can reduce LDL(SMD:0.445 and 0.287 for HIIT and MICT, respectively) but not find in MICT. There was no significant effect of HIIT or MICT on TG, HDL, FBG and Insulin. Comparisons of HIIT and MICT interventions revealed no significant differences in their effects on any measure of body composition, aerobic capacity, lipid and glucose metabolism index(Table 3).

### Subgroup analysis

The results of the subgroup analysis of the effects on outcomes are shown in Table 4. Compared with MICT, subgroup of DHTI ≥ 2 min can significantly increase VO_2max_ (SMD = 0.444, 95% CI: 0.037 ~ 0.851, *P* = 0.032). According to the energy consumption, HIIT were divided into the following subgroups: H = M, H<M, H>M and ?, there are no articles including the subgroup of H>M. Compared with MICT, subgroup of H = M can significantly increase VO_2max_ (SMD = 0.399, 95% CI: 0.106 ~ 0.692, *P* = 0.008). There was no significant difference across subgroups in the mean effect (Table 4).

**Table 4:** Results of subgroup analysis.

Outcome index	Subgroup		Study (n)	SMD (95% CI)	*P*	Weight%	*I*^2^%	Compare across subgroups (*P-*Value)
Weight	DHTI	<2 min	9	0.064(-0.317, 0.445)	0.741	46.46	52.5	0.817
		≥2 min	11	-0.140(-0.630, 0.349)	0.574	53.54	72.0	
	Energy expenditure	H = M	11	-0.219(-0.667, 0.228)	0.337	55.68	71.1	0.693
		H<M	6	0.101(-0.382, 0.584)	0.682	29.90	48.9	
		?	3	0.393(-0.128, 0.913)	0.139	14.42	4.6	
BMI	DHTI	<2 min	6	0.092(-0.634, 0.818)	0.938	37.74	76.4	0.909
		≥2 min	10	-0.028(-0.598, 0.542)	0.924	62.26	76.6	
	Energy expenditure	H = M	10	-0.108(-0.730, 0.513)	0.732	62.90	81.4	0.742
		H<M	3	0.193(-0.503, 0.890)	0.587	25.42	62.1	
		?	2	0.316(-0.390, 1.022)	0.380	11.68	0	
Fat%	DHTI	<2 min	7	-0.147(-0.527, 0.232)	0.447	40.95	39.6	0.998
		≥2 min	11	-0.145(-0.699, 0.408)	0.607	59.05	77.1	
	Energy expenditure	H = M	11	-0.320(-0.818, 0.178)	0.208	61.20	75.7	0.516
		H<M	4	0.189(-0.219, 0.597)	0.363	23.18	0	
		?	3	0.095(-0.454, 0.645)	0.374	15.63	0	
VO_2 max_	DHTI	<2 min	10	0.135(-0.233, 0.504)	0.472	63.97	51.9	0.632
		≥2 min	6	0.444(0.037, 0.851)	0.032*	36.03	26.8	
	Energy expenditure	H = M	10	0.399(0.106, 0.692)	0.008*	64.96	27.5	0.249
		H<M	3	-0.192(-0.957, 0.574)	0.623	18.53	59.4	
		?	3	0.179(-0.613, 0.971)	0.657	16.52	53.7	
TG	DHTI	<2 min	7	-0.057(-0.565, 0.451)	0.827	58.45	60.2	0.893
		≥2 min	5	-0.162(-0.846, 0.522)	0.643	41.55	68.2	
	Energy expenditure	H = M	7	-0.087(-0.548, 0.374)	0.711	59.63	53.8	0.372
		H<M	3	-0.621(-1.443, 0.201)	0.139	24.53	61.5	
		?	2	0.662(-0.246, 1.570)	0.153	15.84	47.8	
TC	DHTI	<2 min	7	-0.098(-0.453, 0.258)	0.590	59.47	22.4	0.334
		≥2 min	5	0.472(-0.084, 1.029)	0.096	40.53	50.8	
	Energy expenditure	H = M	7	0.203(-0.326, 0.733)	0.451	58.41	63.6	0.372
		H<M	3	0.061(-0.419, 0.542)	0.802	25.59	0.0	
		?	2	0.009(-0.967, 0.985)	0.986	16.00	57.8	
LDL	DHTI	<2 min	6	-0.161(-0.495, 0.174)	0.346	71.59	5.4	0.531
		≥2 min	2	0.042(-0.473, 0.557)	0.872	28.41	0.0	
	Energy expenditure	H = M	4	-0.101(-0.480, 0.278)	0.601	55.37	4.5	0.525
		H<M	3	0.094(-0.386, 0.575)	0.700	32.57	0.0	
		?	1	-0.646(-1.436, 0.144)	0.109	12.06	\	
HDL	DHTI	<2 min	7	-0.200(-0.807, 0.408)	0.520	68.46	70.8	0.778
		≥2 min	3	-0.008(-0.468, 0.452)	0.973	31.54	0.0	
	Energy expenditure	H = M	5	0.156(-0.326, 0.639)	0.342	30.86	80.9	0.269
		H<M	3	-0.423(-1.295, 0.449)	0.525	49.19	0.0	
		?	2	0.000(-0.607, 0.607)	1.000	19.96	0.0	
FBG	DHTI	<2 min	6	0.183(-0.605, 0.972)	0.649	61.08	79.2	0.422
		≥2 min	4	-0.726(-2.071, 0.620)	0.290	38.92	87.0	
	Energy expenditure	H = M	6	0.302(-0.238, 0.842)	0.273	61.64	57.7	0.560
		H<M	2	0.009(-1.945, 1.963)	0.993	19.97	87.9	
		?	2	-2.135(-5.104, 0.834)	0.159	18.39	91.9	
Insulin	DHTI	<2 min	5	0.377(-0.292, 1.046)	0.269	69.88	62.0	0.962
		≥2 min	2	0.351(-0.259, 0.961)	0.259	30.12	0.0	
	Energy expenditure	H = M	2	0.792(-1.472, 3.056)	0.493	24.61	89.2	0.054
		H<M	2	0.294(-0.218, 0.806)	0.261	43.78	0.0	
		?	2	0.202(-0.378, 0.783)	0.494	31.61	0.0	

*P<0.05 was defined as a significant difference;

<DHTI: Durations of HIIT training interval; SMD: Standardized mean difference; BMI: Body Mass Index; TG: Triglyceride; TC: Total Cholesterol; LDL: Low-density lipoprotein; HDL: High-density lipoprotein; FBG: Fasting blood glucose. ?:Not Clear; \:There is only one article and *I*^2^% cannot be calculated.

## Discussion

Different exercise modes produce different training effects on body[45]. HIIT and MICT can induce significant improvements in body composition, VO_2max_ and TC. Both HIIT and MICT appear to be similarly effective on risk factors of CVD, however HIIT(30.0±10.2min) save 9.7min compared with MICT(39.7±15.5min) in one session. HIIT is superior to MICT in improving VO_2max_ when durations of HIIT training interval ≥ 2 min or energy expenditure of HIIT same as MICT. Each of these findings has major implications for reducing the risk of CVD management interventions.

### Effects of HIIT and MICT on body composition

The results showed that there were no significant differences between HIIT and MICT in reducing fat%, BMI and weight. Currently, the researches support the idea that HIIT has greater effectiveness on weight loss than MICT[25, 29, 46], indicating that exercise intensity plays an important role in regulation of body composition and local fat consumption [29, 47]. HIIT is superior to MICT in promoting the secretion of catecholamines, epinephrine [48], norepinephrine[49] and growth hormone [50], which promote fat decomposition [51, 52] to achieve effective weight loss. Nevertheless, there is still debate about the effectiveness of HIIT for weight loss and the fat burning mechanism of HIIT[53] because improvement in body composition is affected by exercise intensity, frequency, diet and lifestyle et al[54]. Energy balance is critical in the influence of exercise on body composition, Martins et al.[32] adopted HIIT and MICT in interventions for volunteers with overweight or obese under the same total energy consumption and found no significant differences between HIIT and MICT in reducing fat% and weight, but the exercise time of HIIT was much less than that of MICT. As a result, the consumption of total energy plays a more critical role in weight loss relative to the exercise intensity[55], yet it can also consume large amount of total energy to achieve weight loss through a longer period of MICT [56]. In our study, the duration time of one session of the MICT(39.7±15.5 min) programs is more than that of HIIT(30.0±10.2 min). This review indicates that HIIT may provide similar benefits of improving body composition in less energy expenditure compared with MICT, the benefits of HIIT on fat loss have been proposed to reflect changes in appetite responses and an augmented excess post-exercise oxygen consumption (EPOC)[53], additionally, HIIT has greater potential for muscle glycogen depletion than MICT[19]. However, weight loss management is along-term process, the Institute of Medicine defined weight loss maintenance as losing at least 5% of body weight, or reducing body mass index (BMI) by at least 1 unit, and keeping weight below this minimum amount for at least 1 year[57], either HIIT or MICT implemented in long-term is likely to produce clinically meaningful fat loss. Publication bias has great influence on the stability of fat%, thus more studies on the comparison of fat reduction effects between HIIT and MICT need to be studied in future.

### Effects of HIIT and MICT on VO_2max_

**Fig 2:**
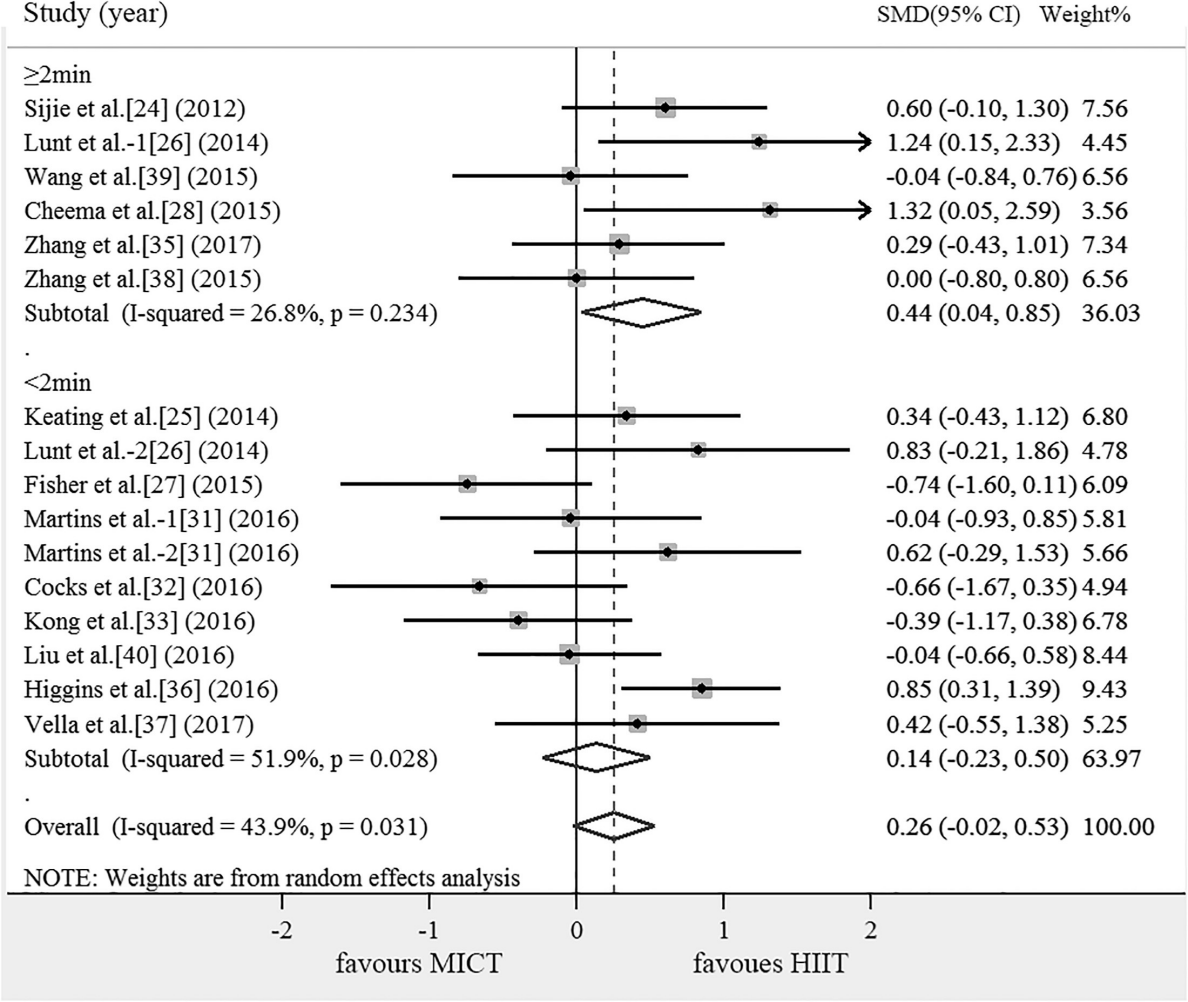
Meta-analysis for the comparison of HIIT vs. MICT for VO_2max_ with subgroup analyses for studies employing DHTI < 2 min, DHTI ≥2 min than MICT. The positive value of X axis indicated that HIIT was more effective than MICT in improving VO_2max_. Lunt et al.-1 and Lunt et al.-2 were two different HIIT methods were used in one article. Martins et al.-1 and Martins et al.-2 were two different HIIT methods were used in one article. DHTI< 2 min: HIIT training interval < 2 min, DHTI ≥2 min: HIIT training interval ≥ 2 min.

**Fig 3:**
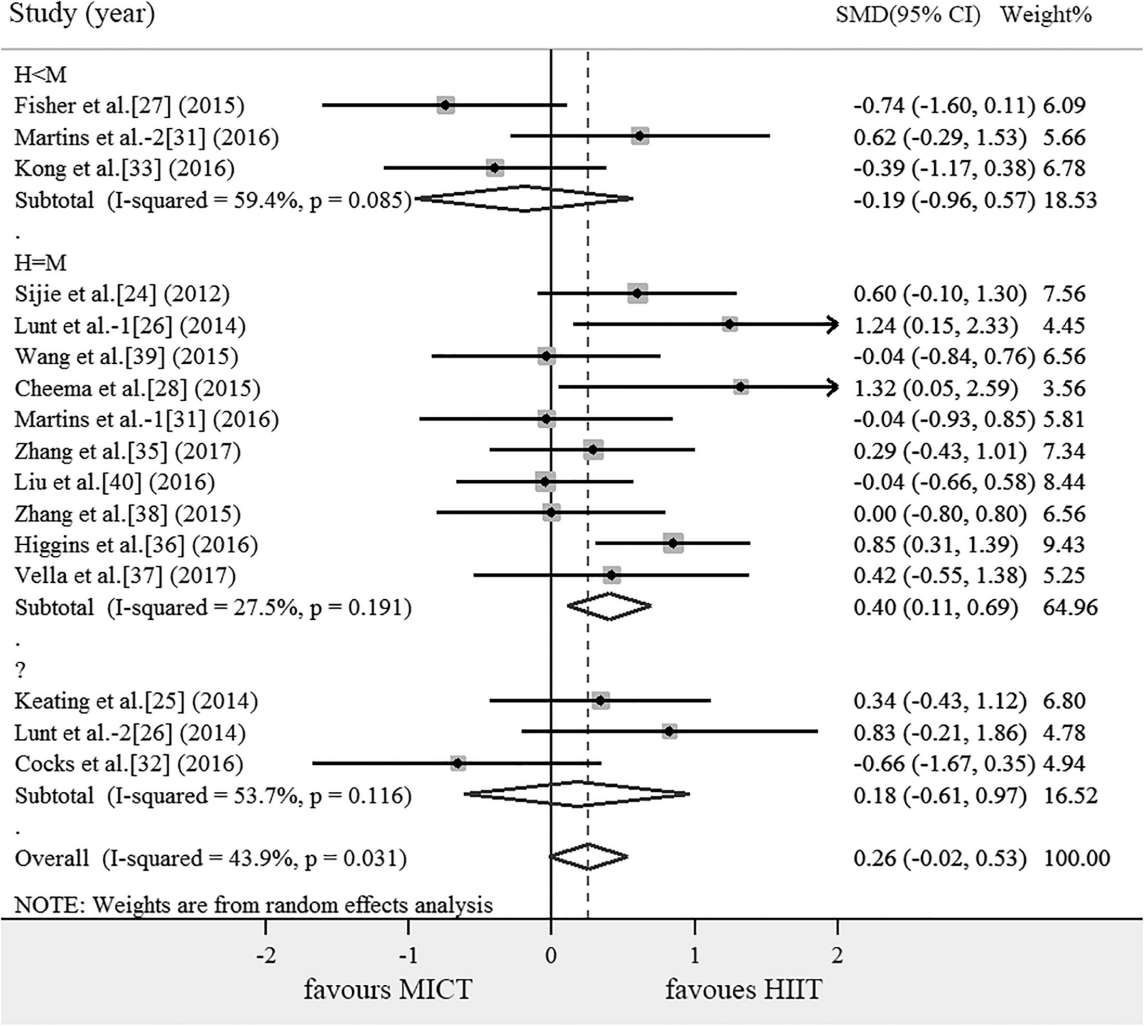
Meta-analysis for the comparison of HIIT vs. MICT for VO_2max_ with subgroup analyses for studies employing H = M, H<M and ? than MICT. The positive value of X axis indicated that HIIT was more effective than MICT in improving VO_2max_. Lunt et al.-1 and Lunt et al.-2 were two different HIIT methods were used in one article. Martins et al.-1 and Martins et al.-2 were two different HIIT methods were used in one article. H = M: Energy expenditure of HIIT equal to MICT;H<M: Energy expenditure of HIIT less than MICT; ?:Not mentioned in the article and the results can not be used to calculate the energy consumption.

In individuals with poor cardiorespiratory fitness, it is much easier to identify CVD than obesity[58]. The risk of CVD decreases by 15% as long as the aerobic capacity increases 1- Metabolic Equivalent of Energy (MET)[59]. Thus, improving cardiorespiratory fitness confers major health benefits for both individuals with obesity and the population with CVD [60]. VO_2max_ is recognized as the important predictors of mortality among cardiac patients, several studies had shown that HIIT was safe in cardiac patients[61], HIIT also can improve VO_2max_ for healthy subjects[62], patients with heart failure[63]. Some researches indicated that HIIT results in greater improvements in VO_2max_ compared to MICT[64, 65], but the results of this review showed that there was no significant difference of HIIT and MICT on improving VO_2max_. Interestingly, subgroup analysis of this study found that DHTI ≥ 2 min had greater effectiveness than MICT, but the effect of DHTI < 2 min on improving cardiorespiratory fitness was similar to that of MICT, which indicates that DHTI ≥ 2 min obtain more advantage in improving VO_2max_(SMD = 0.444, 95% CI: 0.037 ~ 0.851, *P* = 0.032), partly because of DHTI≥ 2 min can keep the heart and lungs in a relatively high working state for a long time. It can be seen that it is necessary to maintain a certain period of time in a higher intensity in order to increase cardiopulmonary fitness. What is the relationship between energy consumption of different sports modes and cardiopulmonary fitness? When compared the energy consumption and the DHTI subgroup, we were surprised to find that 5 of the 6 articles in the DHTI ≥ 2 min subgroup were equal energy expenditure of HIIT and MICT(Figs 2 and 3). It can be seen that energy consumption plays an important role in improving VO_2max_, furthermore, subgroup analysis showed that HIIT (SMD = 0.399, 95% CI: 0.106 ~ 0.692, *P* = 0.008) had an advantage in improving cardiopulmonary fitness with the same energy expenditure as MICT(Fig 3). The possible mechanisms of HIIT to improve cardiorespiratory fitness are as follows: In work-matched comparisons, maximum stroke volume increased more following HIIT relative to MICT, HIIT programme elicited an significant increase in skeletal muscle mitochondrial respiration compared with MICT[66], stroke volume and skeletal muscle mitochondrial respiration enhanced the oxygen utilization rate in peripheral blood, thereby improving VO_2max_ and cardiorespiratory fitness. However HIIT and MICT can achieve similar increases in muscle oxidative capacity, as reflected by the activity of cytochrome c oxidase (COX), COX subunits II, IV protein content[67] and 3-hydroxyacyl CoA dehydrogenase[68]. So both durations of training interval and total exercise load play an important role in improving cardiopulmonary fitness, and HIIT has an advantage in improving cardiopulmonary fitness when energy expenditure of HIIT equal to MICT.

### Effects of HIIT and MICT on lipid metabolism

Individuals with obesity often suffer from lipid metabolism disorder[69], which leads to increase risk of CVD[70]. Exercise is especially known as an economic and effective approach to reduce fat accumulation, however, the results of this review comparisons of HIIT and MICT interventions revealed no significant differences in their effects on TG, TC, LDL, HDL. There was no significant difference in the changes of TC[71], HDL[72], LDL[73], triglycerides[74], and C-reactive protein[75] between HIIT and MICT in the studies examining the improvement of lipid metabolism. The effects of exercise on blood lipid levels in individuals with overweight or obesity depend on blood lipid levels before exercise, exercise intensity, exercise duration, body composition, calorie intake, metabolic rate and lifestyle et al[76–78]. HIIT and MICT in improving TG, TC, LDL, and HDL in adults with overweight and/or obesity may be related to the abovementioned factors, which indicates that these factors should be fully considered in studies examining the influence of exercise mode on blood lipid levels. But the results of this study indicated that HIIT and MICT modes resulted in statistically significant reductions in TC(SMD:0.467 and 0.488 for HIIT and MICT, respectively). Several studies have shown that the decomposition and metabolism of fat have strong sensitivity to hormones[79], and exercise can significantly increase epinephrine, norepinephrine and growth hormone[80] and stimulate fat decomposition[81], the results of this study show that HIIT and MICT are effective approach to reduce TC. Accumulation of LDL inside the blood vessels serves as a major cause of arteriosclerosis, regular exercise enhances the heart and vessel functions and can thus prevent or delay the progress of CVD in part by improving cholesterol levels[82]. This study find that HIIT resulted instatistically significant reductions in LDL(SMD:0.445 and 0.287 for HIIT and MICT, respectively) but not in MICT. Our results suggest that HIIT can prevent LDL accumulation. Regular and continuous exercises especially HIIT help control obesity, reduces the risk of CVD, thus helping prevent or postpone the development of CVD in obese individuals.

### Effects of HIIT and MICT on glucose metabolism

Exercise is an effective way to reduce insulin levels[83], insulin-dependent glucose disposal increases following exercise[84], HIIT tended to be superior in reduction FBG and insulin resistance compared with MICT[18, 85]. Some studies found that HIIT can induce higher expression and translocation of glucose transporter 4 (GLUT4) on skeletal muscle cell membrane surfaces in comparison with MICT[86, 87]. Metcalf et al.[88] noted in an observational study lasting 6 ~ 19 years that the effect of regulating insulin and improving insulin resistance will meaningfully weaken or even disappear without enough exercise intensity and suggested that intermittent exercise of sufficient intensity is needed. Improvement of insulin sensitivity is localized to contracting muscle, HIIT recruit a larger proportion of muscle fibres compared with MICT[89], thus HIIT can improve the efficiency of insulin transportation and insulin sensitivity, thereby reduce blood sugar and inhibiting excessive insulin secretion. However a meta-analysis reported no difference between HIIT and MICT[90]. Tjønna et al[91]. reported a reduction in the FBG of patients with metabolic syndrome after 16 weeks of either continuous exercise or intermittent exercise. Gayda et al.[92] reported that intermittent exercise is as effective as continuous exercise for reducing FBG in patients with chronic heart failure. This study found that there was no significant difference between HIIT and MICT in the improvement of FBG and insulin levels, partly because of the included subjects are overweight and/or obesity adults without metabolic diseases. It is noteworthy that the changes in glucose metabolism with either modality was small in terms of clinical meaningfulness, but, given the health benefits of exercise are fundamental in populations with obesity.

### Limitations

A major limitation of this study is the heterogeneity of the included literature. Although the heterogeneity was reduced by subgroup analysis, some subgroups still had high heterogeneity, which may have affected the reliability of the results. However, this study used the random-effects model to avoid the adverse effects of high heterogeneity. Publication bias has great influence on the stability of fat%. Cardiovascular risk factors involve many aspects, this study was restricted to analyze the aspects of body composition, aerobic capacity and glycolipid metabolism. Hence, the conclusions may be limited.

## Conclusions

HIIT appears to provide similar benefits to MICT for improving body composition, VO_2max_ and TC, but HIIT spent less time than MICT by 9.7 min on one session of training, HIIT can be considered as a time-efficient exercise mode for managing overweight and obese individuals. HIIT resulted instatistically significant reductions in LDL but not in MICT. HIIT is superior to MICT in improving cardiopulmonary fitness when durations of HIIT training interval ≥ 2 min or energy expenditure of HIIT same as MICT.

Prospect: The HIIT mode includes manipulating key programming variables such as frequency, intensity, training interval, recovery interval, the different combinations of these variables will confer different benefits to physical function. Future research directions may include examining which training combinations are more advantageous to improving cardiovascular risk factors.

## Supporting information

S1 File Search strategy. (DOCX)

S1 Checklist PRISMA 2009 checklist. (DOC)

S1 Dataset Revised dataset. (XLSX)
