# Supplementary material for: Effects of HIIT and MICT on cardiovascular risk factors in adults with overweight and/or obesity: A meta-analysis
Source: PLoS One. 2019 Jan 28;14(1):e0210644. doi: 10.1371/journal.pone.0210644 (PMC6349321; doi:10.1371/journal.pone.0210644)
Supplement: S1 File — (DOCX) [file pone.0210644.s001.docx]

The full electronic search strategy for PUBMED：

#1 interval training[TW] OR intermittent exercise[TW] OR interval exercise[TW] OR intermittent training[TW]

#2 Obesity[TW] OR obese[TW] OR overweight[TW]

#3 animals[MeSH Terms] OR adolescent[MeSH Terms] OR child [MeSH Terms]

#1 AND #2 NOT #3
