# Supplementary material for: Effects of HIIT and MICT on cardiovascular risk factors in adults with overweight and/or obesity: A meta-analysis
Source: PLoS One. 2019 Jan 28;14(1):e0210644. doi: 10.1371/journal.pone.0210644 (PMC6349321; doi:10.1371/journal.pone.0210644)
Supplement: S1 Checklist — (DOC) [file pone.0210644.s002.doc]

| **Section/topic** | **#** | **Checklist item** | **Reported on page #** |
| --- | --- | --- | --- |
| **TITLE** | | |  |
| Title | 1 | Effects of HIIT and MICT on cardiovascular risk factors in healthy adults with overweight and/or obesity: a meta-analysis | 1 |
| **ABSTRACT** | | |  |
| Structured summary | 2 | Objective: The purpose of this study was to evaluate the effects of high-intensity interval training (HIIT) and medium-intensity continuous training (MICT) on cardiovascular disease (CVD) risk factors in healthy adults with overweight and obesity. Methods: Twenty-one articles were included by searching databases PubMed, et al. the total number of samples was 606 in these articles. Outcomes were synthesised using a random-effects meta-analysis of the weighted mean difference (WMD) in CVD risk factors. Results: HIIT is more effective in reducing fat% (WMD=-0.434, P=0.029) and increasing VO2max (WMD=0.775, P=0.038) than MICT in healthy adults with overweight and/or obesity. Durations of HIIT training interval ≥ 2 min (DHTI ≥ 2 min) was more effective in improving VO2max (WMD=1.213, P=0.039) and reducing fasting blood glucose (FBG) (WMD=-0.291, P< 0.001) than MICT, DHTI ≥ 2 min was marginally effective in reducing fat% (WMD=-0.516, P=0.073) comparing with MICT. MICT was marginally better than DHTI ≥ 2 min in improving TC (WMD=7.171, P=0.082). Conclusions: HIIT is more effective in reducing fat% and increasing VO2max than MICT in healthy adults with overweight and/or obesity. DHTI ≥ 2 min is more advantageous in reducing fat%, FBG and improving VO2max than MICT, but less advantageous in reducing TC.This research program has been registered on the PROSPERO System Evaluation Registration Platform, registration number: CRD42016045835. | 2 |
| **INTRODUCTION** | | |  |
| Rationale | 3 | People who develop CVD not only bear extreme suffering leading to declines in quality of life but also pose a heavy economic burden to their families and the society. Therefore, it is of practical importance to identify an appropriate method, especially anon-drug method, of improving the risk factors of CVD and reducing its incidence. Moderate intensity continuous training (MICT) is often considered an effective way to reduce risk factors of CVD. However, in recent years, a popular method called high intensity interval training (HIIT) characterized by lower exercise frequency and shorter exercise time has also been shown to be effective in reducing risk factors of CVD. | 2 |
| Objectives | 4 | we aimed to quantitatively combine data from experimental studies comparing the effect between HIIT and MICT in reducing risk factors of CVD in healthy adults with overweight and/or obesity. Findings from this study may serve important evidence for prevention CVD in healthy adults with overweight and/or obesity. | 3 |
| **METHODS** | | |  |
| Protocol and registration | 5 | Indicate if a review protocol exists, if and where it can be accessed (e.g., Web address), and, if available, provide registration information including registration number. |  |
| Eligibility criteria | 6 | Inclusion criteria were as follows: (a) randomized controlled trial (RCT) or controlled clinical trial (CCT); (b) healthy participants ≥ 18 years old, BMI ≥ 25; (c) HIIT intervention and MICT intervention; (d) ≥ 4 weeks with intervention; (e) outcome indicators included at least one of the following: weight, body mass index (BMI), fat mass% (fat%), VO2max, triglyceride (TG), total cholesterol (TC), low-density lipoprotein (LDL), high-density lipoprotein (HDL), fasting blood glucose (FBG) and insulin; (f) the study reported that baseline BMI of HIIT participants or baseline BMI could be calculated from the provided data; (g) the article was written in English or Chinese.  Exclusion criteria were as follows: (a) participants with chronic diseases, such as diabetes, hypertension, or other diseases constituting a special population; (b) studies on one-time acute exercise; (c) interventions including strength training, diet or medicine; (d) HIIT without supervision. | 4 |
| Information sources | 7 | The searched databases included PubMed, Embase, Cochrane, CENTRAL, PEDro and CNKI. The publication dates of the articles were restricted to the period between the year when the database was built to the present. The last search date was July 20, 2018. | 3 |
| Search | 8 | Keywords such as “high-intensity interval training”, “high-intensity interval exercise”, “high-intensity intermittent exercise”, “anaerobic interval exercise”, “sprint interval exercise”, HIIT, HIT, HIIE, SIT, overweight, obesity, and obese were searched. | 3 |
| Study selection | 9 | Two authors independently screened titles and abstracts of potentially eligible studies and downloaded the full texts. | 4 |
| Data collection process | 10 | Discrepancies between the two authors during the literature exclusion were solved through reaching consensus with each other. Another way of obtaining eligible studies was through examining the references of relevant studies. | 4 |
| Data items | 11 | All statistical calculations were performed by statistical software STATA 14.1 (Release 14.1 College Station, TX, USA); P ≤ 0.05 was defined as a significant difference, and 0.5 < P ≤ 0.1 was defined as a marginally significant difference. | 6 |
| Risk of bias in individual studies | 12 | The quality of the literature was evaluated according to the risk bias evaluation method adopted by Costigan et al. in a meta-analysis of HIIT-related research. The eight evaluation items were as follows: (a) inclusion criteria; (b) randomized grouping; (c) baseline similarity; (d) rater-masked; (e) intentional analysis; (f) participants’ withdrawal proportion was less than 20%; (g) the sample quantity met the requirements; (h) accurate results were reported. The two researchers used “√” (with clear description), “×” (without description) and “?” (unknown or inadequate description) to evaluate each included article. The inconsistent results were solved through discussion among the research group, and each article with a “√” was counted. | 4 |
| Summary measures | 13 | Weight, BMI, fat%, VO_2max_, TG, TC, LDL, HDL, FBG, Insulin | 4 |
| Synthesis of results | 14 | The statistical heterogeneity was examined using I2 between included studies and Cochran’s Q-test. It was defined as non-existent, low, medium and high heterogeneity when I2 values were < 25%, 25 ~ < 50%, 50 ~ < 75%, and ≥ 75%, respectively. Egger’s test was adopted to detect publication bias. Furthermore, to test the reliability of the results of this study, the following two methods were used to conduct a sensitivity analysis: (a) Fixed-effects models replaced the random-effects models to count the results again; and (b) one article was removed each time to examine whether each article had a significant influence on the effect. | 5 |

Page 1 of 2

| **Section/topic** | **#** | **Checklist item** | **Reported on page #** |
| --- | --- | --- | --- |
| Risk of bias across studies | 15 | Table 2 Risk of bias assessment of included studies | 11 |
| Additional analyses | 16 | According to the sensitivity analysis of the 21 articles, if statistical models were replaced and one article was removed each time to perform a meta-analysis again, the results of effect would not change meaningfully, indicating that the results of the meta-analysis in this study were reliable.HIIT must be applied appropriately by manipulating key programming variables (frequency, intensity, training interval, recovery interval). There are difference contributions of the anaerobic energy systems during events of differing training interval and intensities. The phosphagen and fast glycolytic are the primary energy systems when durations of HIIT training interval < 2 min (DHTI < 2 min), the fast glycolytic energy systems will be gradually depleted when durations of HIIT training interval ≥ 2 min (DHTI ≥2 min). HIIT programmes were divided into the following subgroups: DHTI ≥ 2 min and DHTI < 2 min. | 10 and 15 |
| **RESULTS** | | |  |
| Study selection | 17 | A total of 305 articles were searched from each database; 284 articles were excluded according to the inclusion and exclusion criteria, and 21 articles were included. The literature screening process is shown in Fig 1. | 6 |
| Study characteristics | 18 | There were 606 participants in the 21 articles examined in this study. A total of 303 samples were in the HIIT group, with 6 ~ 29 samples in each group; a total of 303 samples were in the MICT group, with 6 ~ 29 samples in each group. The training frequency was 3 ~ 5 times a week, and the cycle was 4 ~ 12 weeks. The participants included in this study were trained by ergometry cycle [18, 20, 22, 25-28, 30-32, 35], walking, jogging and running [16, 19, 21, 23, 24, 29, 33, 34, 36, 37]. The following monitoring methods were used in the MICT group: 60 ~ 75% HR_max_ [17, 20, 25, 26, 29], 50 ~ 60% VO_2max_[19, 24, 30, 35-37], 10% below the individual anaerobic threshold (IAT) intensity [18], 400% MET (metabolic equivalent of energy) [23], 60 ~ 80 VO_2peak_ [20, 22, 27, 28], 60 ~ 75% HR_peak_ [34] and 55 ~ 70%HRR[4, 32]. The following monitoring methods were used in HIIT group: 85 ~ 95%HR_max_ [17, 21, 23, 25, 29], 85 ~ 90% VO_2max_ [19, 24, 30, 35-37], all out[21, 26, 28, 31-33, 35], 85% peak power [22], 85 ~ 95% HR_peak_ [34], 20% above the IAT intensity[18], 200% W_max_ [27] and 120% VO_2peak_ [20]. VO_2max_ was determined by the method of the stepwise increasing load test combined with gas metabolic analysis. The standards of the end of exercise were that the respiratory quotient was more than 1.05 [17, 26], 1.1 [28, 32, 34] or 1.15 [19, 21, 24], combined with subjective consciousness and the exhaustion of willpower[23, 25, 30]. Blood collection after fasting 8 ~ 12 hours was needed for biochemical index detection. The methods adopted for the body composition test included the following: dual X-ray[17, 19, 20, 22, 25-27, 30, 31], bioelectrical impedance [18, 24, 28, 34-37], air displacement plethysmography with measured thoracic gas volume[32], and skin fold calculation[23]. The outcome indexes included body composition (weight, BMI, fat%), VO_2max_ and glycolipid metabolism indicators (TG, TC, LDL, HDL, FBG, insulin). The included studies' characteristics are shown in Table 1. | 6-7 |
| Risk of bias within studies | 19 | Of the 21 articles, 5 articles were classified as low risk (7 ~ 8), 15 articles were classified as moderate risk (4 ~ 6), and one article was classified as high risk (0 ~ 3), Risk of bias assessment of included studies are presented in Table 2. According to the sensitivity analysis of the 21 articles, if statistical models were replaced and one article was removed each time to perform a meta-analysis again, the results of effect would not change meaningfully, indicating that the results of the meta-analysis in this study were reliable. | 10 |
| Results of individual studies | 20 | Table 3 Effects of HIIT vs. MICT on Body composition, Aerobic capacity, Lipid metabolism, Glucose metabolism   | Category | Index | Study  (n) | WMD (95% CI) | *P* | Heterogeneity | | Egger’s test *P* | | --- | --- | --- | --- | --- | --- | --- | --- | | *I^2^*(%) | *P* | | Body composition | Weight, kg | 19 | -0.134 (-0.647, 0.379) | 0.610 | 67.7 | 0.000 | 0.892 | | BMI, kg/m^2^ | 15 | 0.005 (-0.235, 0.245) | 0.965 | 80.4 | 0.000 | 0.593 | | fat% | 17 | -0.434 (-0.824, 0.044) | **0.029***^*^* | 49.4 | 0.011 | 0.073 | | Aerobic capacity | VO_2max_,  ml/kg/min | 16 | 0.775 (-0.042, 1.509) | **0.038***^*^* | 49.4 | 0.013 | 0.507 | | Lipid metabolism index | TG, mg/dl | 12 | -2.506 (-11.085, 6.072) | 0.567 | 69.8 | 0.000 | 0.447 | | TC, mg/dl | 12 | 2.359 (-2.489, 7.207) | 0.340 | 56.5 | 0.008 | 0.646 | | LDL, mg/dl | 8 | -1.422 (-6.019, 3.175) | 0.544 | 0.0 | 0.487 | 0.345 | | HDL, mg/dl | 19 | -1.490 (-5.220, 2.349) | 0.434 | 84.2 | 0.000 | 0.684 | | Glucose metabolism index | FBG, mmol/l | 10 | -0.012(-0.225, 0.250) | 0.918 | 87.1 | 0.000 | 0.117 | | Insulin, mU/l | 7 | 0.980 (-0.426, 2.386) | 0.172 | 70.5 | 0.002 | 0.282 |   *^*^P*≤0.05 was defined as a significant difference; ^†^0.5<*P*≤0.1 was defined as a marginally significant difference; MICT: medium-intensity continuous training; HIIT: high-intensity interval training; WMD: weighted mean difference; BMI: Body Mass Index; TG: triglyceride; TC: Total Cholesterol; LDL: Low-density lipoprotein; HDL: High-density lipoprotein; FBG: fasting blood glucose. | 14 |
| Synthesis of results | 21 | The results of effect sizes of comparisons of HIIT and MICT are shown in table 3. Results for meta-analysis of body composition: Differences between HIIT and MICT were observed for fat% (WMD = -0.434, 95% CI: -0.824 ~ 0.044, *P* = 0.029) (Fig 2), with moderate heterogeneity across studies (*I^2^* = 49.4%, *P* = 0.011), but there was no significant differences between HIIT and MICT in weight (WMD = -0.134, 95% CI: -0.647 ~ 0.379, *P* = 0.610), BMI (WMD = 0.005, 95% CI: -0.235 ~ 0.245, *P* = 0.965) in healthy adults with overweight and/or obesity (Table 3). Results for meta-analysis of VO_2max_: HIIT had a significant increase in VO_2max_ (WMD = 0.775, 95% CI: -0.042 ~ 1.509, *P* = 0.038) comparing with MICT in healthy adults with overweight and/or obesity, with moderate heterogeneity across studies (*I^2^* = 49.4%, *P* = 0.013) (Fig 3). Results for meta-analysis of glycolipid metabolism: There were no significant differences between HIIT and MICT in TG (WMD = -2.506, 95% CI: -11.085 ~ 6.072, *P* = 0.567), TC (WMD = 2.359, 95% CI: -2.489 ~ 7.207, *P* = 0.340), LDL (WMD = -1.422, 95% CI: -6.019 ~ 3.175, *P* = 0.544), HDL (WMD = -1.490, 95% CI: -5.220 ~ 2.349, *P* = 0.434), FBG (WMD = -0.012, 95% CI: -0.225 ~ 0.250, *P* = 0.918) and insulin (WMD = 0.980, 95% CI: -0.426 ~ 2.386, *P* = 0.172)in healthy adults with overweight and/or obesity. | 12 |
| Risk of bias across studies | 22 | Of the 21 articles, 5 articles were classified as low risk (7 ~ 8), 15 articles were classified as moderate risk (4 ~ 6), and one article was classified as high risk (0 ~ 3), Risk of bias assessment of included studies are presented in Table 2. | 10 |
| Additional analysis | 23 | HIIT programmes were divided into the following subgroups: DHTI ≥ 2 min and DHTI < 2 min. The results of the subgroup analysis of the effects on outcomes are shown in Table 4. Compared with MICT, subgroup of DHTI ≥ 2 min can significantly increase VO_2max_ (WMD = 1.213, 95% CI: 0.062 ~ 2.364, *P* = 0.039), lower FBG (WMD = -0.291, 95% CI: -0.361 ~ 0.222, *P* = 0.000) and marginally reduce fat% (WMD = -0.516, 95% CI: -1.081 ~ 0.049, *P* = **0.073**), but DHTI ≥ 2 min is less advantageous (marginally difference) than MICT with respect to improving TC (WMD = 7.171, 95% CI: -0.915 ~ 15.257, *P* = 0.082). | 15 |
| **DISCUSSION** | | |  |
| Summary of evidence | 24 | (a) HIIT saves time, is highly efficient in improving cardiovascular risk factors and has some advantages over MICT in reducing fat% and increasing VO_2max_ in healthy adults with overweight and/or obesity;  (b) This study for the first time divided HIIT programmes into two subgroups: DHTI ≥ 2 min and DHTI < 2 min subgroups. DHTI ≥ 2 min is more advantageous in improving VO_2max_ and reducing fat% and FBG than MICT;  (c) The effect of HIIT vs. MICT on lipid metabolism is not significantly different, but subgroup analysis indicate that DHTI ≥ 2 min is less advantageous (marginally difference) than MICT with respect to decreasing TC. | 21 |
| Limitations | 25 | A major limitation of this study is the heterogeneity of the included literature. Although the heterogeneity was reduced by subgroup analysis, some subgroups still had high heterogeneity, which may have affected the reliability of the results. However, this study used the random-effects model to avoid the adverse effects of high heterogeneity, thus improving the reliability of the results. Cardiovascular risk factors involve many aspects, this study was restricted to analyze the aspects of body composition, aerobic capacity and glycolipid metabolism. Hence, the conclusions may be limited. | 20 |
| Conclusions | 26 | DHTI ≥ 2 min has advantages for improving fat%, VO2max and FBG. According to this conclusion, an efficient exercise mode can be selected based on an individual’s cardiovascular risk situation, which also provides the basis for making personalized exercise recommendations for individuals with overweight or obese. The HIIT mode includes manipulating key programming variables such as frequency, intensity, training interval, recovery interval, the different combinations of these variables will confer different benefits to physical function. Future research directions may include examining which training combinations are more advantageous to improving cardiovascular risk factors. | 21 |
| **FUNDING** | | |  |
| Funding | 27 | |  | | --- |   This study was supported by the National Natural Science Foundation of China (81703252, to MingHui Quan). |  |

*From:*  Moher D, Liberati A, Tetzlaff J, Altman DG, The PRISMA Group (2009). Preferred Reporting Items for Systematic Reviews and Meta-Analyses: The PRISMA Statement. PLoS Med 6(7): e1000097. doi:10.1371/journal.pmed1000097

For more information, visit: **www.prisma-statement.org**.

Page 2 of 2
